# Adaptive Value Normalization in the Prefrontal Cortex Is Reduced by Memory Load

**DOI:** 10.1523/ENEURO.0365-17.2017

**Published:** 2017-04-27

**Authors:** L. Holper, L. D. Van Brussel, L. Schmidt, S. Schulthess, C. J. Burke, K. Louie, E. Seifritz, P. N. Tobler

**Affiliations:** 1 Department of Psychiatry Psychotherapy and Psychosomatics, Psychiatric Hospital, University of Zurich, 8032 Zurich, Switzerland; 2 Laboratory for Social and Neural Systems Research, Department of Economics, University of Zurich, 8006 Zurich, Switzerland; 3 Center for Neural Science, New York University, New York, NY 10003; 4 Institute for the Interdisciplinary Study of Decision Making, New York University, Brooklyn, NY 11201

**Keywords:** adaptive coding, value normalization, risky decision-making, memory load, prefrontal cortex, computational model comparison, functional near-infrared spectroscopy

## Abstract

Adaptation facilitates neural representation of a wide range of diverse inputs, including reward values. Adaptive value coding typically relies on contextual information either obtained from the environment or retrieved from and maintained in memory. However, it is unknown whether having to retrieve and maintain context information modulates the brain’s capacity for value adaptation. To address this issue, we measured hemodynamic responses of the prefrontal cortex (PFC) in two studies on risky decision-making. In each trial, healthy human subjects chose between a risky and a safe alternative; half of the participants had to remember the risky alternatives, whereas for the other half they were presented visually. The value of safe alternatives varied across trials. PFC responses adapted to contextual risk information, with steeper coding of safe alternative value in lower-risk contexts. Importantly, this adaptation depended on working memory load, such that response functions relating PFC activity to safe values were steeper with presented versus remembered risk. An independent second study replicated the findings of the first study and showed that similar slope reductions also arose when memory maintenance demands were increased with a secondary working memory task. Formal model comparison showed that a divisive normalization model fitted effects of both risk context and working memory demands on PFC activity better than alternative models of value adaptation, and revealed that reduced suppression of background activity was the critical parameter impairing normalization with increased memory maintenance demand. Our findings suggest that mnemonic processes can constrain normalization of neural value representations.

## Significance Statement

The influence of mnemonic processes on value-based decision-making is only beginning to be understood. In two separate studies, we investigate how having to maintain information in working memory affects efficient and adaptive value coding in lateral prefrontal cortex during risky decisions. We show that the neural suppression of background-related activity, which allows for efficient and adaptive value coding without working memory demands, is reduced with higher working memory load. Our findings suggest that working memory load can constrain the normalization of neural value representations, illuminating a novel facet of the interplay between working memory and value-based decision-making.

## Introduction

Neural systems need to encode vast ranges of inputs, but the limited dynamic range of neural activities potentially limits fine discriminations between similar inputs (Fairhall et al., 2001; Ollerenshaw et al., 2014). Adaptive coding is a mechanism that facilitates neural discrimination between different inputs by taking advantage of information contained within a given context. It works by dynamically adjusting the sensitivity of the neural apparatus to the most likely inputs in a given context. Adaptive coding was first described for sensory inputs, but evidence now suggests that it also extends to value-related signals, including subjective stimulus values, outcome values, and prediction errors (Rangel and Clithero, 2012; Louie et al., 2015; animal work: Tremblay and Schultz, 1999; Tobler et al., 2005; Kennerley and Wallis, 2009; Padoa-Schioppa, 2009; Kobayashi et al., 2010; Louie et al., 2011; Rushworth et al., 2011; Cai and Padoa-Schioppa, 2012; human work: Akitsuki et al., 2003; Nieuwenhuis et al., 2005; Elliott et al., 2008; Palminteri et al. 2015; Park et al., 2012; Cox and Kable, 2014; Burke et al., 2016; Kirschner et al., 2016).

Context-dependent adaptation of neural sensitivity to value can be described by several competing models. These models have been developed originally for the visual system and concur on the notion that context modulates neural responding to given stimuli but disagree on how exactly this modulation is implemented. For example, divisive normalization (Heeger, 1992; Heeger et al., 1996; Carandini et al., 1997) states that activity of a visual neuron reflects the input from its receptive field (e.g., a preferred stimulus inside the receptive field) normalized by the summed activity of a pool of neighboring neurons (e.g., the set of all other nonpreferred stimuli outside the receptive field). By contrast, a nondivisive alternative model proposes that neighboring context activity is subtracted from receptive field activity. Importantly, adaptation mechanisms are thought to extend to value coding processes, with the adaptation models capturing the activity of value-processing regions (Louie et al., 2015). For example, neurons coding value positively may respond more strongly to higher magnitudes of safe alternatives (i.e., preferred stimuli) but may be suppressed when less valuable, e.g., risky alternatives (i.e., nonpreferred stimuli), are presented simultaneously as a possible choice option.

Typically, adaptive coding is studied with adaptation-inducing contexts being physically present. However, in real life, these contexts often have to be remembered and maintained in working memory, for example during investment decisions that incorporate previously encoded information about the financial state of a company. Yet, it is largely unknown how adaptive value coding is affected by having to maintain information in working memory. To address this question, we investigated neural value adaptation in two studies with two experiments each. In Study 1, we varied working memory demand only by requiring participants to memorize the contextual choice alternative or not, whereas in Study 2, we additionally varied working memory load in a secondary task independent of context. Specifically, both studies consisted of two different risky decision experiments: in the dual-alternative experiments, a safe and a risky choice alternative were presented simultaneously, whereas in the single-alternative experiments, only a safe alternative was presented, and the risky alternative had to be maintained in working memory. In Study 2, participants in addition had to maintain either a sequence of numbers and letters (high working memory load) or a sequence of zeros (low working memory load). We hypothesized that the absence of risky alternatives and high working memory load would reduce the capacity for adaptive coding owing to a higher background activity induced by the higher demand on working memory. This prediction is compatible with a full divisive normalization model (Louie et al., 2011), according to which higher baseline pooled activity codetermines adaptation. Given that neurons in the prefrontal cortex (PFC) show sustained activity due to higher working memory demands (Levy and Goldman-Rakic, 2000; Lara and Wallis, 2015), we expected that normalization-based background activity would be higher in the single- than the dual-alternative experiments (Studies 1 and 2) and in high than low working memory load conditions (Study 2). This would result in reduced capacity of divisive normalization as expressed in flatter response functions in the single- compared with the dual-alternative experiments and in high compared with low working memory load conditions.

## Materials and Methods

We performed two studies. In Study 1, we assessed the effects of context-related working memory demands on adaptive value coding in the single-alternative experiment compared with the memory-free dual-alternative experiment. In Study 2, we manipulated working memory demand also in a context-independent fashion by varying the amount of information participants had to maintain in a secondary task while they performed either the single-alternative experiment or the dual-alternative experiment.

### Study 1

#### Participants

For Study 1, 41 healthy participants (22 females, mean age ± SD = 22.8 ± 2.1 years) were recruited through the Registration Center for Study Participants of the University of Zurich. Participants were randomly assigned to perform either in the dual-alternative experiment (*n* = 21, 13 females, mean age = 22.6 ± 2.3 years) or in the single-alternative experiment (*n* = 20, 9 females, mean age = 22.9 ± 1.8 years). All participants were right-handed (mean laterality quotient LQ = 88.7 ± 21.4) according to the Edinburgh Handedness Inventory (Oldfield, 1971). Exclusion criteria were any psychiatric or neurologic disorders or current medication. All participants gave written informed consent. The study was approved by the ethics committee of the Canton Zurich and performed in accordance with the Declaration of Helsinki.

#### Experimental protocol

All participants completed a variant of a risky-decision task in which they were asked to make repeated choices between safe and risky alternatives (Christopoulos et al., 2009; Tobler et al., 2009; Holper et al., 2014). The task was implemented in Matlab (Version 2014a; Mathworks), and stimulus delivery was controlled using Cogent 2000 (Wellcome Department of Imaging Neuroscience, London, UK). In the present work, we focused on signals elicited by the safe alternatives as processed at the onset of choices between safe and risky alternatives, regardless of which alternative was chosen.

**Figure 1.**
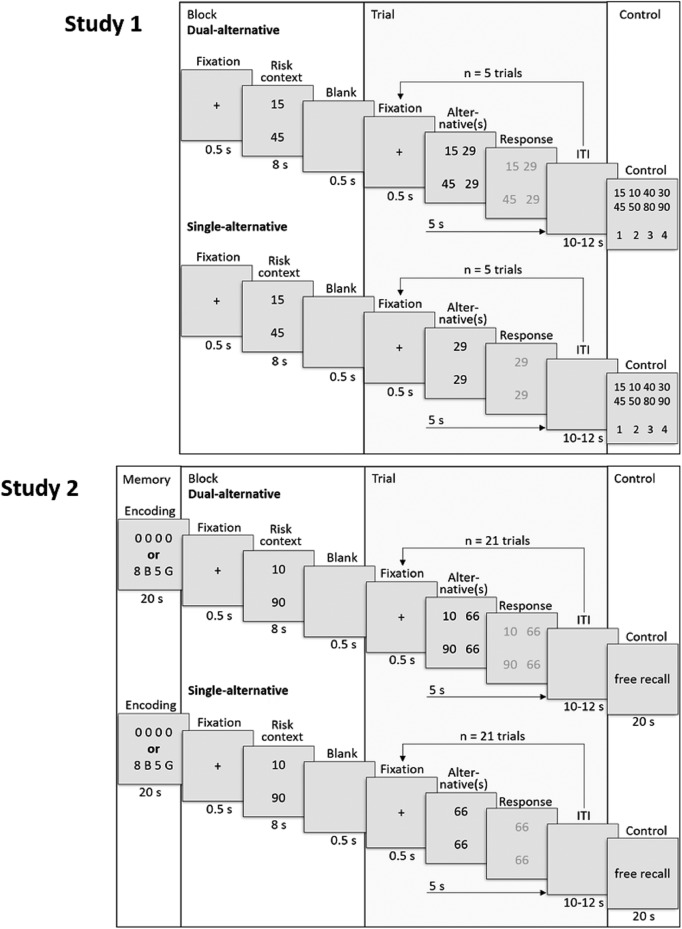
Experimental design. Top: Study 1. Trial structure of the dual- and single-alternative experiment. The block part consisted of the context-setting presentation of the risky alternative and was identical for both experiments. By contrast, the trial part differed between experiments in that both the risky and the safe alternative were shown in the trials of the dual-alternative experiment, whereas only the safe alternative was shown in the trials of the single-alternative experiment. In the control part, participants indicated which risky alternative was active during the preceding block by pressing the corresponding number key on the keyboard. Bottom: Study 2. Trial structure was as in Study 1, but each block was preceded by a secondary working memory part, represented by a context-independent sequence of numbers and letters, that had to be maintained in working memory and recalled from longer-term memory at the end of the block.

Both the dual-alternative and the single-alternative experiment consisted of eight blocks of five trials, resulting in a total of 40 trials (Fig. 1, top). In both experiments, safe alternatives varied from trial to trial, whereas risky alternatives varied only across blocks. When referring to the original normalization model (Louie et al., 2011) with in- and outside receptive field inputs, we therefore treated the safe values as the varying inside (preferred) stimuli, whereas the risky alternatives were entered as the more constant outside (nonpreferred) stimuli. Safe alternatives indicated that participants would receive the given outcome for sure (100%), whereas risky alternatives indicated an even-chance probability (50/50%) for two possible outcomes. Please note that risk here is defined as variance, following the definition of finance theory (Markowitz, 1952). All alternatives were presented in Swiss francs (CHF). Four risky alternatives were used, such that two risk levels were crossed with two levels of expected values (EV). In particular, there were two low-risk levels with either low EV (CHF 15/45) or high EV (CHF 40/80), and two high-risk levels with either low EV (CHF 10/50) or high EV (CHF 30/90). The two risk levels served to manipulate adaptive coding. Safe alternatives were determined semirandomly within the range of the risky alternative of a given block, ensuring that experienced within-block EV approximated across-block EV for each of the four risky alternatives.

Each block started with a fixation cross shown for 0.5 s, followed by an 8-s presentation of the risky alternative to be used in this block. Participants were instructed to remember the risky alternative. After the risky alternative, a blank screen was shown for 0.5 s, followed by the presentation of five choice-trials involving that risky alternative. The instructions and trial structure were common to both the dual- and the single-alternative experiment. Participants were allowed to practice the task before the start of the experiment.

##### Dual-alternative experiment

In the dual-alternative experiment, safe and risky alternatives were presented simultaneously in each of the five trials. For a period of 5 s, the safe and risky alternatives were each presented on one side of the screen. Participants were instructed to make their choices through key press within these 5 s. Once choices were made, both alternatives slightly changed color to indicate that the response was recorded. Participants were not informed about the outcome of their choices to prevent learning and to control for the possibility that outcomes would influence subsequent behavior or brain activity. If participants failed to respond in time, a brief message instructed them to “please respond faster.” Missed trials were excluded from the analysis. Trials were separated by an intertrial interval with a variable duration of 10–12 s, drawn from a uniform distribution.

##### Single-alternative experiment

The single-alternative experiment differed from the dual-alternative experiment in that only the safe alternatives were presented throughout the five trials. Thus, in each trial, only one safe alternative was presented in the middle of the screen, therefore requiring participants to remember the risky alternative. Again, participants chose the safe or risky alternative by pressing a key on the keyboard and safe alternatives varied from trial to trial.

In both the dual- and single-alternative experiment, at the end of each block, participants were asked to indicate which of the four possible risky alternatives had been available for choice during the block. This control question checked whether participants remembered the risky alternative used throughout the five trials of the block. One participant who gave random answers to these control questions was excluded from the analysis. Other than that, participants made no mistakes. At the end of the complete experimental session, participants received the outcome of one randomly drawn trial (in CHF). If the draw resulted in a trial in which participants had chosen a risky alternative, the outcome of the 50/50% gamble was determined by a random Boolean, where 0 indicated the lower of the two outcomes and 1 indicated the higher of the two outcomes.

### Study 2

#### Participants

Study 2 was conducted in a different set of participants than Study 1. It included 11 participants in the dual-alternative experiment (5 females, mean age = 24.5 ± 3.7 years) and 12 participants in the single-alternative experiment (7 females, mean age = 25.8 ± 5.8 years).

#### Experimental protocol

Study 1 revealed impaired neural adaptation in the single-alternative compared with the dual-alternative experiment. In line with this finding, neural adaptation effects with single-alternatives are not unprecedented (Tobler et al., 2005; Kirschner et al., 2016). However, adaptation studies often use multiple stimuli or alternatives. In Study 2, we therefore tested whether the more unusual presentation format of the single-alternative experiment might explain the effects or whether increasing working memory load through an independent secondary task also would increase baseline activity in the PFC (see “Hemodynamic responses” in Results) and reduce neural value adaptation in the more typical dual-alternative layout.

In Study 2, we therefore varied working memory load separately of the single-alternative versus dual-alternative manipulation. Specifically, we redesigned three aspects of Study 1 (Fig. 1). First, we added a secondary, decision-independent working memory part before both dual- and single-alternative experiments. This additional, context-independent, working memory load also translated more closely the idea that visual adaptation effects arise from the interplay between preferred (context-dependent) and nonpreferred (context-independent) receptive field stimulation, although the analogy should be interpreted with care. Second, we used a finer scale of the safe alternatives by assessing 21 safe alternatives for each risky alternative (instead of 10 alternatives in Study 1). The finer scale of the safe alternatives allowed for more complete sampling and better qualitative assessment of value adaptation. Third, we increased working memory load by requiring participants to remember the risky alternative over 21 trials (instead of 5 trials in Study 1). We hypothesized that both context-dependent and context-independent working memory load will be evidenced in PFC. If both types of working memory load affect neural adaptation in a similar manner, then adding high context-independent working memory load to the dual-alternative experiment with the secondary task may have effects on neural adaptation similar to removing one choice alternative in single-alternative experiments.

The addition of low or high context-independent working memory load was implemented by the presentation of a sequence of items at the start of each block in both the dual- and single-alternative experiment. In low-load blocks, we presented a sequence of zeros (i.e., 0 0 0 0), whereas in high-load blocks, we presented a sequence of numbers and letters (e.g., 8 B 5 G; Fig. 1, bottom). In both the dual- and single-alternative experiments, at the end of each block, participants were asked to reproduce the sequence that had been presented at the start of the block by pressing the corresponding numbers and letters on the keyboard. This control question checked whether participants remembered the sequence throughout the 21 trials of each block. Two participants (one from the dual- and one from the single-alternative experiment) who made more than two sequence recall mistakes overall were excluded from further analysis.

### Instrumentation

**Figure 2.**
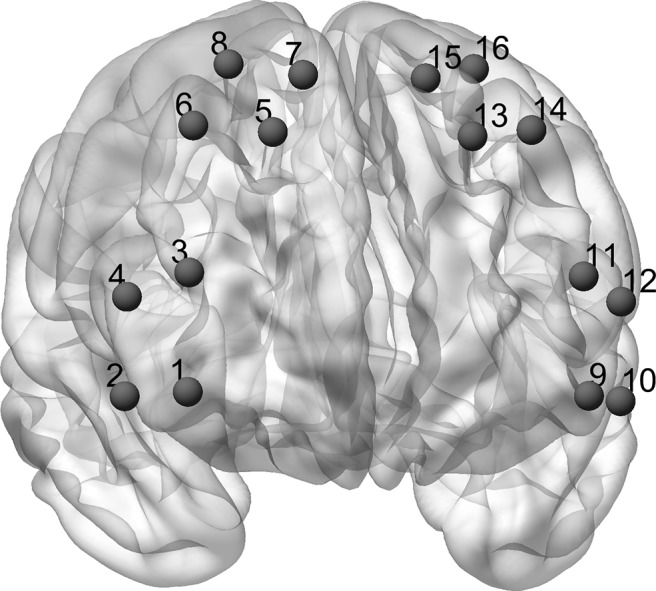
fNIRS channel positions. The channel setup covered parts of the lateral and medial PFC. For final analysis, we averaged over all channels. The Matlab toolbox NFRI (Singh et al., 2005) was used to estimate the Montreal Neurologic Institute coordinates corresponding to the international EEG 10–20 positions. Channel positions were visualized using BrainNet Viewer (Xia et al., 2013).

In both studies, hemodynamic responses were recorded using functional near-infrared spectroscopy (fNIRS; LLC NIRx Medical Technologies). 16 channels covered parts of the lateral and medial PFC (see Fig. 2 for channel positions). The system used time-multiplexed dual-wavelength light-emitting diodes with wavelengths of 760 and 850 nm. Photoelectrical detectors containing silicon photodiodes (Siemens) were used for optical recording. Light sources and detectors were placed in a flexible head cap to allow for direct skin contact (Epitex). The source-detector geometry ensured a distance of ∼30 mm between each source and detector. The data acquisition board was connected to a notebook computer running NIRStar 14.0 (LLC NIRx Medical Technologies). The sampling rate was 7.81 Hz.

The NIRSLab analysis software (Xu et al., 2014) was used to preprocess the functional recordings, including baseline corrections, and removal of long-term trends. NIRSlab was also used to detect and remove motion artifacts, which was done in 23 (study 1) and 5 (study 2) participants after visual inspection. Changes in total hemoglobin (Δ[tHb]), derived as the sum of changes in oxygenated (Δ[O_2_Hb]) and deoxygenated (Δ[HHb]) hemoglobin, was chosen as the primary parameter of interest. Δ[tHb] represents changes in blood volume correlated with blood flow (Grubb et al., 1974) and is presumed to be relatively insensitive to vein contamination, thus providing higher spatial specificity for mapping cerebral activity compared with Δ[O_2_Hb] or Δ[HHb] separately (Gagnon et al., 2012).

To compute Δ[tHb] estimates, we used the general linear model approach (Uga et al., 2014) with an adapted hemodynamic response function (HRF) from the onset of alternatives for 40 sliding time intervals (partially overlapping). Briefly, the adapted HRF differs from the standard functional MRI (fMRI) approach of using GLMs together with a canonical HRF in that the optimum temporal parameters of the peak delay of the HRF were systematically changed to determine the best-fitting model for the underlying O_2_Hb and HHb time series data. Time intervals were 2.5 s long and separated by 180 ms. Thus, together the intervals covered the hemodynamic activity over 8 s after onset of the alternatives. This interval-based computation was chosen to characterize the adaptive value responses with high temporal resolution. Generally, the peak of the hemodynamic response is assumed to occur at 5–6 s after stimulus onset, as reported by previous fNIRS-based studies (Jasdzewski et al., 2003; Matthews et al., 2008; Cui et al., 2010; Hoshi, 2011).

## Data Analysis

The analyses described here examined the adaptation of prefrontal hemodynamic responses to the values of the safe alternatives, under (1) the four different contexts (i.e., the risky alternatives), (2) the dual- versus single-alternative experiment (i.e., the presence versus the absence of the risky alternatives), and (3) the high versus low context-independent working memory load in Study 2. All statistical analyses were performed using Matlab.

### Response time and choice behavior

To examine behavioral effects of working memory load and risk contexts, response times (RTs) were calculated from the onset of the choice alternatives to the time of the participant’s button press (“Alternatives” to “Response” in Fig. 1). Moreover, choice behavior was assessed as percentage of trials in which participants selected the risky alternative (% risky choice). For both RT and % risky choice in Study 1, we assessed the steepness of the slopes with regard to the safe values using linear regression on the level of single participants and compared the regression slope coefficients using ANOVA with the within-subject factors *risk* and *EV*, and the between-subject factor *experiment*, including the Bonferroni correction for multiple comparisons on the group level; for Study 2, we added the within-subject factor *load*. The results are reported at a significance level of *p* < 0.05.

### Hemodynamic responses

To identify adaptive coding, we assessed the steepness of the Δ[tHb] slopes using linear regression on the level of single participants and compared regression slope coefficients using ANOVA with the within-subject factor *context* and the between-subject factor *experiment*, including the Bonferroni correction for multiple comparisons on the group level. The results were reported at a significance level of *p* < 0.05.

### Model predictions

To examine how well-known models of adaptive coding captured the hemodynamic responses in the PFC, the Δ[tHb] beta estimates were fitted to two linear and two nonlinear models of value adaptation previously considered by Louie et al. (2011). Note that compared with the original publication (Louie et al., 2011), we renamed and changed the order of models 1 and 2 to clarify their relations and differences.

The difference model (model 1) is a nondivisive model of adaptation. It predicts that neural activity (*A*) follows a linear function of the difference between the safe alternative values and the risky alternative values:

(1) $$A=a+b\;(V_{safe}-V_{risky}),$$

where *V*_safe_ are the safe alternative values, *V_risky_* are the risky alternatives, and *a* and *b* are fitted individual intercept and scaling parameters. To obtain one value for each risk context, *V_risky_* we calculated the standard deviations of the four possible risky alternatives (low risk and low EV: 15/45; low risk and high EV: 40/80; high risk and low EV: 10/50; high risk and high EV: 30/90, in units points).

The basic divisive normalization model (model 2) predicts that *A* is captured by a linear function of the safe alternative values *V*_safe_ normalized by the total sum of the safe and risky alternative values *V_risky_*:

(2) $$A=a+b\;\frac{V_{safe}}{V_{safe}+V_{risky}}.$$

The enhanced divisive normalization model (model 3) predicts that *A* follows a nonlinear function of the safe alternative values *V*_safe_ and the risky alternative values *V_risky_*, with two additional parameters, where *A_max_* is the maximum pooled activity, i.e., the maximum amplitude of the averaged PFC signal, and sigma (σ) is the response slope, i.e., the activity level at which *A_max_* reaches half the amplitude:

(3) $$A=A_{max}\;\frac{V_{safe}}{\sigma +V_{safe}+V_{risky}}.$$

The full divisive normalization model (model 4) is similar to the enhanced divisive normalization model but includes an additional parameter beta (β) representing baseline pooled activity, i.e., the baseline amplitude of the averaged PFC signal:

(4) $$A=A_{max}\;\frac{V_{safe}+\beta }{\sigma +V_{safe}+V_{risky}}.$$

The relation between the three parameters can be summarized by the ratio (*A_max_* * β/σ) representing the level of predicted background activity (*A_Background_*). Background activity (*A_Background_*) is here defined as the overall resulting activity, taking into account *A_max_*, σ, and baseline pooled activity β. Background activity thereby also represents the activity when no stimuli were presented while participants fixated on the fixation cross during the intertrial interval. Generally, *A_max_*controls the amplitude of the response, whereas σ and β control the steepness of the slopes. Note that at large values relative to σ (*V* ≫ σ), divisive normalization approaches a basic form of the model as σ becomes negligible. According to our hypothesis stated in the introduction, we expected that *A_max_*and σ, representing maximum pooled activity and response slope, would be the critical parameters determining context-dependent differences, whereas the parameter β, representing baseline pooled activity, would be the critical parameter determining between-experiment differences.

Models 1 and 2, i.e., the difference model and the basic divisive normalization model, are linear models coding absolute value, whereas models 3 and 4, i.e., the enhanced and the full divisive normalization model, are nonlinear models representing context-dependent value coding. Following our hypothesis that baseline pooled activity relates the effects of working memory load to adaptive coding and given that baseline activity only features in the full normalization model, we expected this model to outperform the other models in explaining the between-experiment differences.

Each of the four models was fitted to the Δ[tHb] data using either simple linear regression (models 1 and 2) or nonlinear regression (models 3 and 4). The models were fitted separately for each channel and each time interval of the Δ[tHb] data, per context, experiment, and participant. In addition, a separate analysis was performed over all data to obtain an overall Akaike information criterion (AIC). The AIC from the overall data analysis was then used to compare the goodness of fit to the various models. The AIC provides an information theoretic basis for model comparison that considers both goodness of fit and parsimony (Akaike, 1974). No significant differences in model fit were found between the 16 prefrontal channels (all differences between ΔAIC per participant as assessed using *t* test were *p* > 0.05), and we therefore averaged over all channels.

To examine the predictive power of the models, we tested the model fits for each participant separately against the rest of the sample using leave-one-participant-out cross-validation, to form an average measure of fit. Based on this, the root mean squared error (RMSE) between the model predictions and the actual data was calculated for each model.

Finally, we computed Pearson correlations between the Δ[tHb] data and each of the four model predictions as a secondary measure of model fit following Louie et al. (2011). To assess the differences between correlation coefficients, we applied the two-sample Kolmogorov–Smirnov test, separately for the two experiments, and illustrated the correlation differences using kernel smoothing function estimates, which show the distribution of correlation coefficients in a more continuous format than histograms.

## Results

We first describe the results of Study 1 in detail (Figs. 1–7) and then provide the findings for Study 2 (Fig. 8).

### Response time and choice behavior

We assessed both RT and choice behavior with regard to between-context and between-experiment differences by comparing the regression slope coefficients on the group level using an ANOVA with the within-subject factor *context* and the between-subject factor *experiment*.

**Figure 3.**
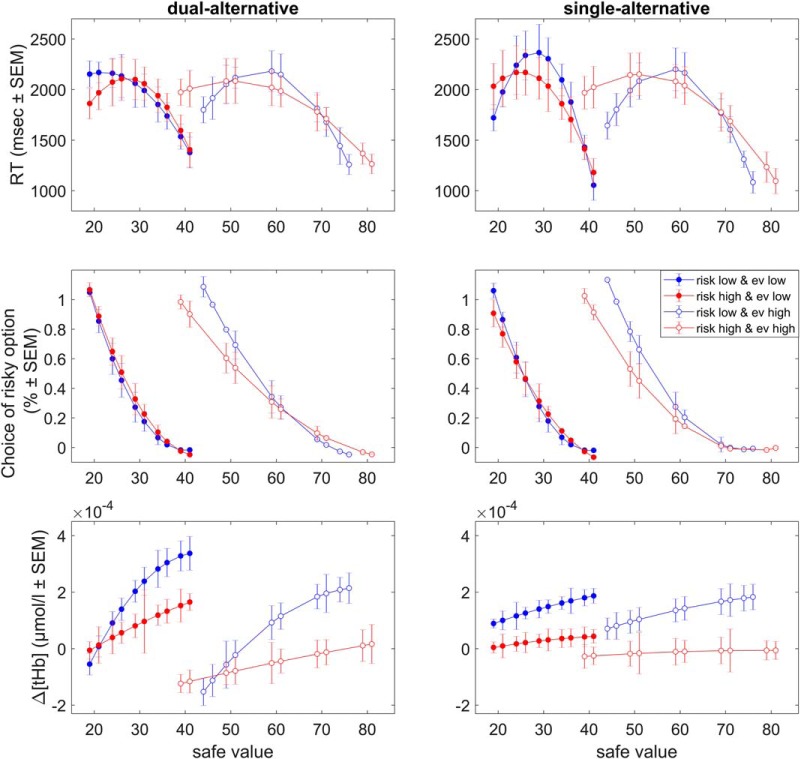
Behavior and basic neural responses in Study 1. Top: RT slopes. Plots illustrate the effects of risk context and working memory load on RT, separately for the dual- and single-alternative experiments. (Middle) Choice slopes. Plots illustrate the effects of context and working memory load on choices of risky options, separately for the dual- and single-alternative experiments. Bottom: Δ[tHb] slopes. Plots illustrate the context- and experiment-dependence of Δ[tHb] response slopes averaged over all channels, separately for the dual- and single-alternative experiments. Slopes are shown for the peak response (time interval 4–7 s after alternatives onset). Note that all slopes are shown in terms of the coefficients for the second polynomial that best fitted the data (in a least-squares sense). Error bars represent SEM. See Table 1 for statistics.

RT decreased with increasing safe alternative values, indicating faster RT with larger safe values, and showed a quadratic effect reflecting choice difficulty (Fig. 3, top). There was a small effect of *context* (*F* = 2.677, *p* = 0.049; however, without significant *post hoc* comparisons; Table 1, RT) but no effect of *experiment* (*F* = 0.884, *p* = 0.345) and no interaction (*F* = 1.117, *p* = 0.344). Together, these results showed that RT were slightly context-dependent, but not affected by working memory load.

**Table 1.**
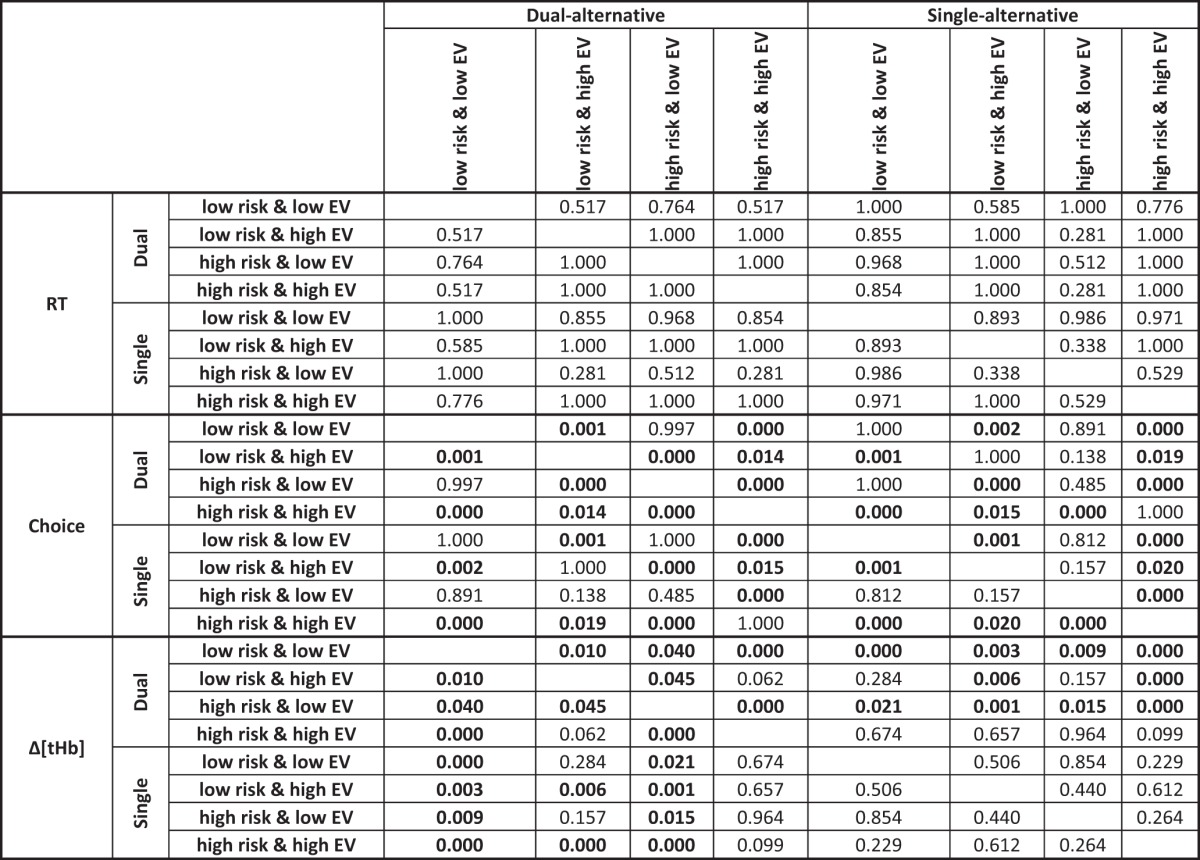
P-values of slope differences, Study 1

P-values context-dependent differences (top left/bottom right quadrants of groups of 8 rows and 8 columns) and dual-alternative vs. single-alternative experiment differences (top right/bottom left quadrants) in regression coefficients relating RT, choice, and Δ[tHb] to safe values in each context, assessed using ANOVA with Bonferroni correction on the group level. Significant differences are bold (*p* < 0.05). See Fig. 3 for illustration.

Choice behavior also showed a decreasing relationship with increasing safe alternative values, indicating fewer risky choices with larger safe values (Fig. 3, middle; Table 1, Choice). Again, there was an effect of *context* (*F* = 48.491, *p* < 0.001), but no effect of *experiment* (*F* = 0.755, *p* = 0.386) and no interaction (*F* = 1.087, *p* = 0.356). *Post hoc* comparisons revealed more steeply decreasing slopes in the low risk and low EV contexts compared with high EV contexts (low risk and high EV and high risk and high EV), in both experiments (dual- and single-alternative). Together, these results showed that choice behavior was context dependent, but not affected by working memory load.

### Hemodynamic responses

To illustrate the neural responses, we averaged Δ[tHb] data over the time interval 4–7 s after the onset of the alternatives. Responses showed a linearly increasing relationship with increasing safe alternative values (Fig. 3, bottom). Thus, prefrontal responses increased with safe values as shown previously with fMRI (Tobler et al., 2009).

To identify adaptive coding, we tested whether the steepness of the slopes was context dependent and/or experiment dependent and found both to be true. We found an effect of *context* (*F* = 7.622, *p* < 0.001), an effect of *experiment* (*F* = 28.024, *p* < 0.001), and an interaction (*F* = 2.619, *p* = 0.048). In particular, low-risk contexts (low risk and low EV, low risk and high EV) and the dual-alternative experiment resulted in steeper slopes compared with high-risk contexts (high risk and low EV, high risk and high EV) and the single-alternative experiment.

Similar to the behavioral data, we assessed the between-context and between-experiment differences by comparing the regression slope coefficients on the group-level using an ANOVA with the within-subject factor *context* and the between-subject factor *experiment*, including Bonferroni correction for multiple comparisons (Table 1, Δ[tHb]). Low-risk contexts showed significantly steeper response increases compared with high-risk contexts, with the between-context differences being significant in the dual-alternative experiment but not in the single-alternative experiment (Table 1). Thus, in the dual-alternative experiment, responses to safe values increased more when participants considered a low-risk alternative, in line with context-dependent adaptive coding of reward value. Further, the dual-alternative experiment showed significantly steeper slopes than the single-alternative experiment (Table 1), suggesting that adaptation was significantly reduced in the single-alternative case. Together, these results showed that neural adaptation of prefrontal value signals was both context dependent and affected by working memory load.

### Model predictions

**Figure 4.**
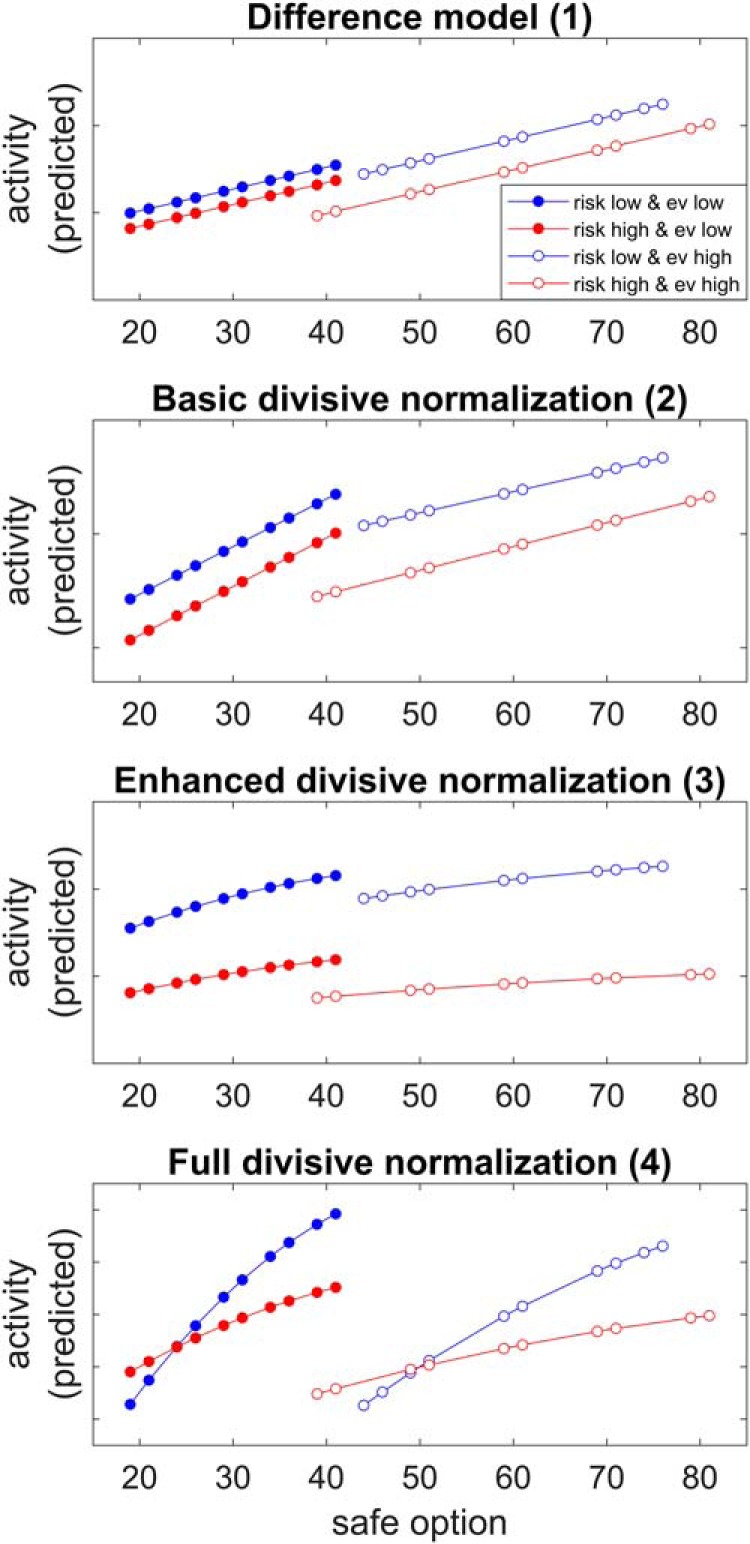
Adaptive coding predicted by different adaptive coding models. Shown are predictions of neural activation in the dual-alternative experiment, based on theoretical parameter values as adapted from the models described by Louie et al. (2011), i.e., the difference model (model 1), the basic divisive normalization model (model 2), the enhanced divisive normalization model (model 3), and the full divisive normalization model (model 4).

To examine whether different models of adaptive coding could explain the adaptation of prefrontal responses during risky decisions, we compared how well the four models predicted hemodynamic responses in the dual- and the single-alternative experiments. Fig. 4 illustrates theoretical data (using assumed parameter values) predicted by the four models of value normalization captured in Eqs. 1–4 (Louie et al., 2011). The figure specifically demonstrates the different predicted response slopes as a function of the safe alternative values for the difference model (model 1), the basic divisive normalization model (model 2), the enhanced divisive normalization model (model 3), and the full divisive normalization model (model 4). Furthermore, because we expected and found steeper slopes under low-risk compared with high-risk contexts, the figure illustrates model 4 for cases with lower β values, i.e., representing lower baseline pooled activity, under low-risk compared with high-risk contexts. Importantly, only models 3 and 4 predicted adaptation to risk contexts and, at least based on visual inspection, the predictions of model 4 appeared to match the observed data (Fig. 3) particularly well for the dual-alternative experiment.

### Model comparison between experiments

**Figure 5.**
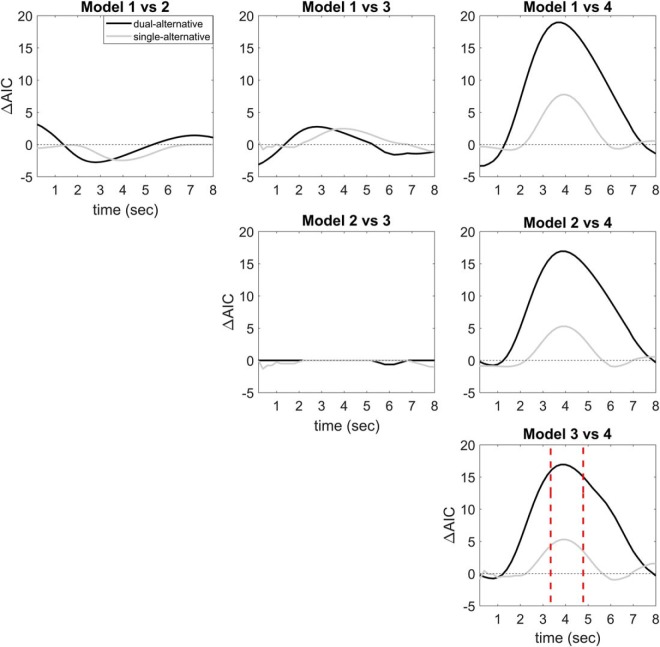
Model comparison. Plots illustrating the time-resolved AIC-differences (ΔAIC) averaged over all channels between models 1–4, separately for the two experiments. Positive values correspond to a relatively better fit of the last-named model. Model 4 (full divisive normalization model) showed a relatively better fit compared with the other models, i.e., the difference model (model 1), the basic divisive normalization model (model 2), and the enhanced divisive normalization model (model 3). Moreover, model 4 provided a better overall fit around the canonical hemodynamic response peak (4 s) in the dual- compared with the single-alternative experiment.

Each of the models was fitted to the Δ[tHb] response separately for each channel, time interval, context, experiment, and participant. The difference in the AIC values (ΔAIC) was calculated for each model pair to compare the relative goodness of fit. No significant differences in ΔAIC were found between the 16 prefrontal channels, and we therefore averaged over all channels. Considering the 40 sliding time intervals, the fit was highest at ∼4 s after the onset of the choice alternatives. Model 4 (full divisive normalization model) overall showed the best fit to the data in both experiments (Fig. 5, third column). Furthermore, model 4 fitted the data of the dual-alternative experiment relatively better than those of the single-alternative experiment (Fig. 5, third row).

To corroborate these findings, we examined the predictive power of the models. Of the four different models, the full divisive normalization model produced the lowest RMSE in prediction, for both the dual- and the single-alternative experiments (RMSE_full_norm_: dual-alternative = 0.0039, single-alternative = 0.0038, *t* = 1.342, *p* = 0.224). The next best model was the enhanced divisive model, which generated slightly higher RMSE, followed by the basic and the difference model (RMSE_enhanced_norm_: dual-alternative = 0.0046, single-alternative = 0.0041, *t* = 3.231, *p* = 0.034; RMSE_basic_norm_: dual-alternative = 0.0056, single-alternative = 0.0052, *t* = 3.001, *p* = 0.028; RMSE_diff_: dual-alternative = 0.0056, single-alternative = 0.0055, *t* = 1.67, *p* = 0.321). In accordance with the ΔAIC results, the smaller RMSEs suggested that the full divisive normalization model (model 4) outperformed the other models in terms of predictive power.

Finally, we asked whether recent trial history may account for our findings, because it is known that reward adaptation can develop over time (Padoa-Schioppa, 2009; Kobayashi et al., 2010). To address this possibility, we repeated the analysis of the current trial data while covarying out the previous trial condition. Again, we found an advantage of the full divisive normalization model over all the other models, as well as a relatively better fit for the dual-alternative experiment compared with the single-alternative experiment (data not shown for succinctness). Thus, recent trial history could not account for our findings.

### Model correlation between experiments

Pearson product-moment correlation coefficients served as secondary measures of model fit to evaluate the relation between each of the four models and the hemodynamic data. We assessed the differences between the correlation coefficients in the two experiments using two-sample Kolmogorov–Smirnov tests and illustrated them using kernel smoothing function estimates. No significant differences were found between the correlation coefficients of the contexts (low risk and low EV, high risk and low EV, low risk and high EV, high risk and high EV); therefore results were averaged over all contexts.

**Figure 6.**
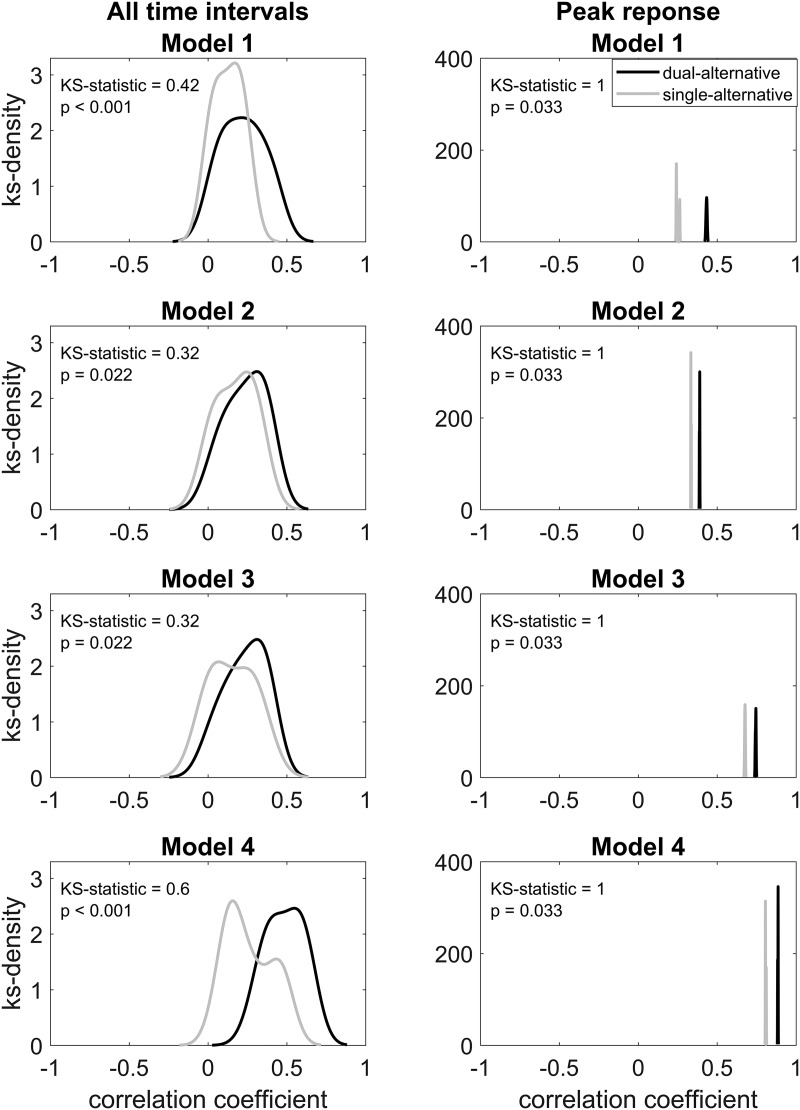
Correlation between models and observed data. Kernel smoothing function estimate (KS-density) of the correlation coefficients between the four predicted models and the observed data in the two experiments. Significant differences between the dual- and single-alternative experiments were assessed using two-sample Kolmogorov–Smirnov test (KS-statistic, *p* value), considering all 40 sliding time intervals (left) and only the peak response (right).

As expected, model 4 (full divisive normalization model) overall correlated more strongly with the observed data in both experiments compared with models 1–3 (difference model, basic divisive normalization model, and enhanced divisive normalization model; Fig. 6, left). Considering the comparison between experiments, the dual-alternative experiment data correlated more strongly with model 4 compared with the single-alternative experiment data (over all time intervals: *p* < 0.001; peak response, 4 s after alternatives onset: *p* = 0.033; Fig. 6, right). Together, the data supported our previous observations that the full divisive normalization model (model 4) fitted best to the hemodynamic responses, and supported that working memory load reduced model fit.

### Model parameters

**Figure 7.**
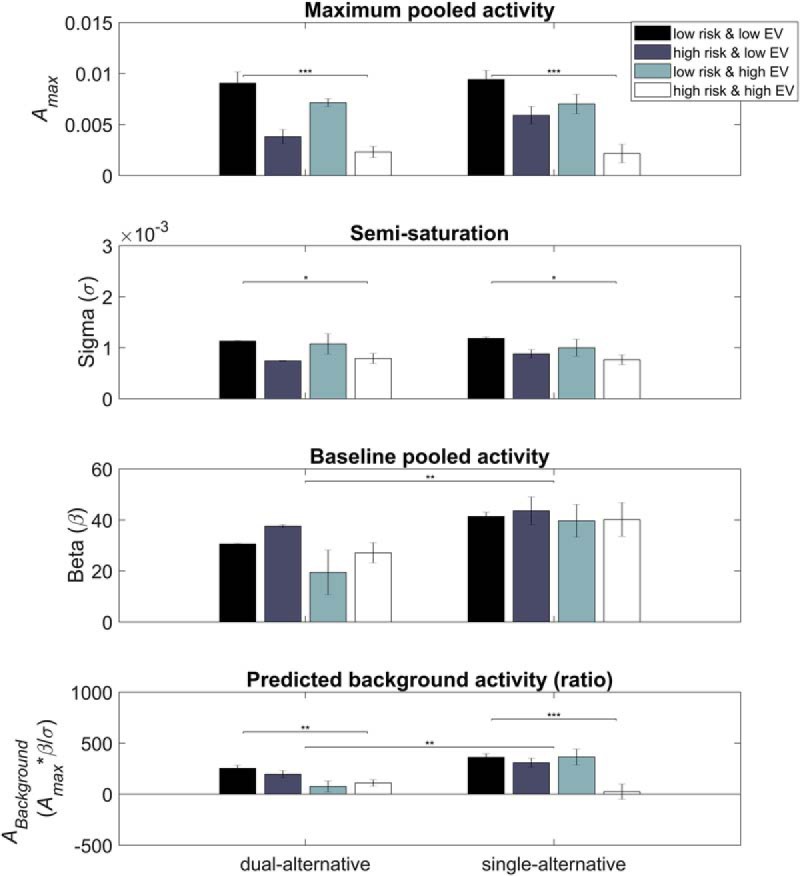
Model parameters. We estimated the parameters for model 4, where *A_max_* was the maximum activity, σ was the response slope, and β was the baseline parameter. The ratio (*A_max_* * β/σ) of the three parameters can be summarized as the predicted background activity (*A_Background_*). See Table 2 for statistical analysis using ANOVA.

As described in Eq. 4, the full divisive normalization model (model 4) involved three parameters, the maximum activity *A_max_*, the response slope σ, and the baseline activity β. To analyze the fitted parameters of model 4 in more detail, their context- and experiment-dependent differences were assessed with an ANOVA (Fig. 7; Table 2). For this analysis, all regressions were run separately for each context.

**Table 2.**
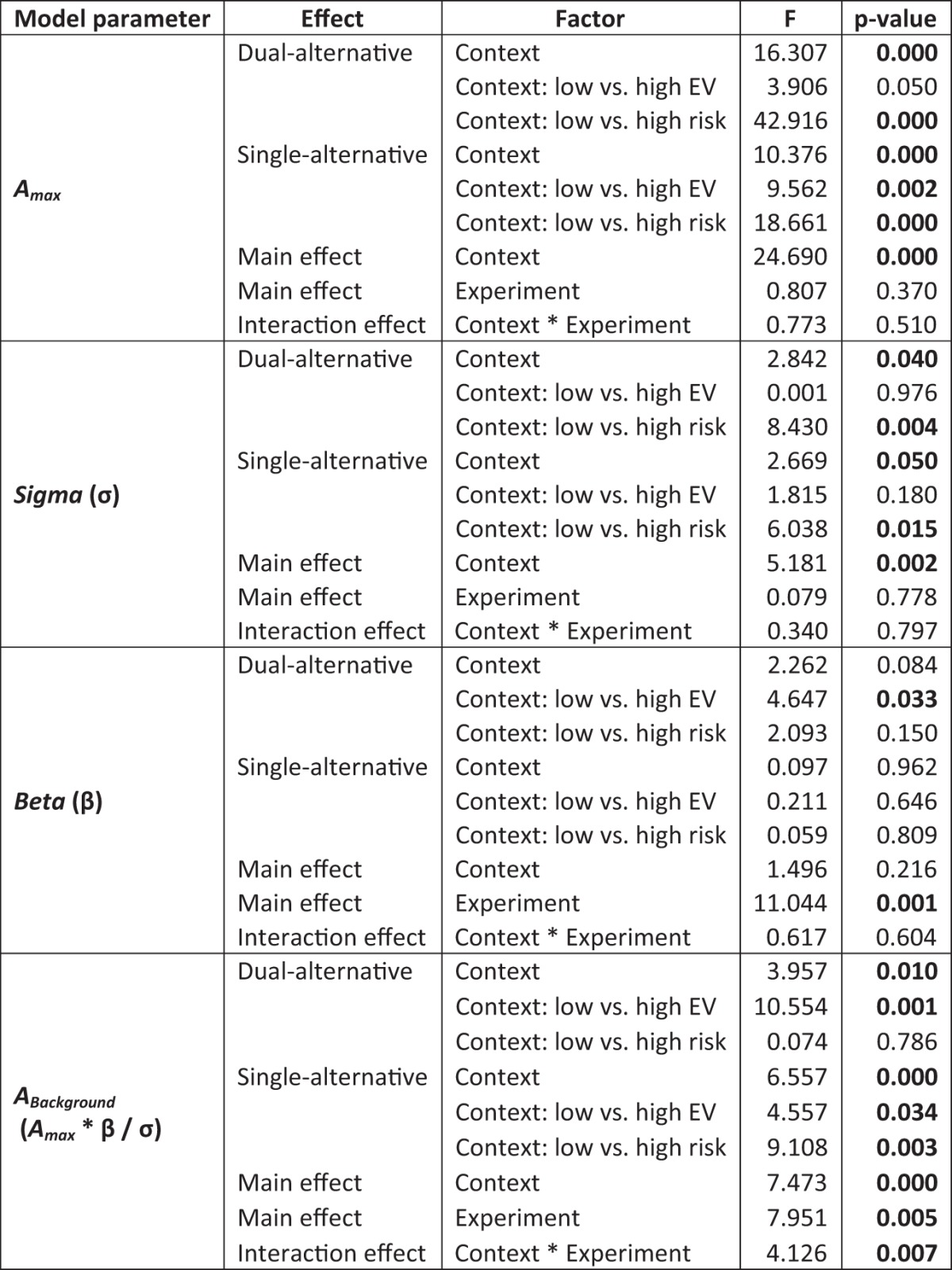
Comparison of model parameters, Study 1

ANOVA using the within-subject factor *context* and the between-subject factor *experiment* to assess the differences in the model parameters estimated in model 4, where *A_max_* was the maximum activity, σ was the response slope, and β was the baseline parameter. The ratio (*a_max_* * β/σ) of the three parameters can be summarized as the predicted background activity (*A_Background_*). Significant differences are bold (*p* < 0.05). See Fig. 7 for illustration.

Regarding the maximum activity (*A_max_*) parameter, results showed context-dependent effects (main effect of *context*, *F* = 24.690, *p* < 0.001) in both the dual-alternative (*F* = 16.307, *p* < 0.001) and single-alternative (*F* = 10.376, *p* = 0.001) experiments. There were no significant differences between experiments or an interaction of experiment with context (main effect of *experiment F* = 0.807, *p* = 0.370; interaction *context* * *experiment*, *F* = 0.773, *p* = 0.510). Regarding the response slope parameter σ, results were similar, with context-dependent effects (main effect of *context*, *F* = 5.181, *p* = 0.002) in both the dual-alternative (*F* = 2.842, *p* = 0.040) and single-alternative (*F* = 2.669, *p* = 0.049) experiments, but no effects involving experiments (main effect of *experiment*, *F* = 0.079, *p* = 0.778; interaction *context* * *experiment*, *F* = 0.340, *p* = 0.797). Together, these results suggest that greater maximal activity (*A_max_*) and steeper response slopes (σ) explained the context-dependent hemodynamic differences in low- versus high**-**risk contexts.

Regarding the β parameter, results showed no context-dependent effects (main effect of *context*, *F* = 1.496, *p* = 0.216; dual-alternative, *F* = 2.262, *p* = 0.084; single-alternative, *F* = 0.097, *p* = 0.962). However, there was a significant difference between experiments indicating a lower baseline parameter in the dual- compared with single-alternative experiment (main effect of *experiment*, *F* = 11.044, *p* = 0.001; interaction *context* * *experiment*, *F* = 0.617, *p* = 0.604). Thus, differences in baseline pooled activity explained hemodynamic differences between experiments.

Taking all parameters together, these effects resulted in a significant context- and experiment-dependent difference of the predicted background activity (*A_Background_*) as represented by the ratio (*A_max_* * β**/**σ; main effect of *context*, *F* = 7.473, *p* < 0.001; main effect of *experiment*, *F* = 7.951, *p* = 0.005; interaction *context* * *experiment*, *F* = 4.126, *p* = 0.007). Thus, the suppression of background activity (with baseline pooled activity being the critical parameter) was significantly reduced in the single- compared with the dual-alternative experiment, which reduced the capacity for divisive normalization in PFC.

### Study 2

**Figure 8.**
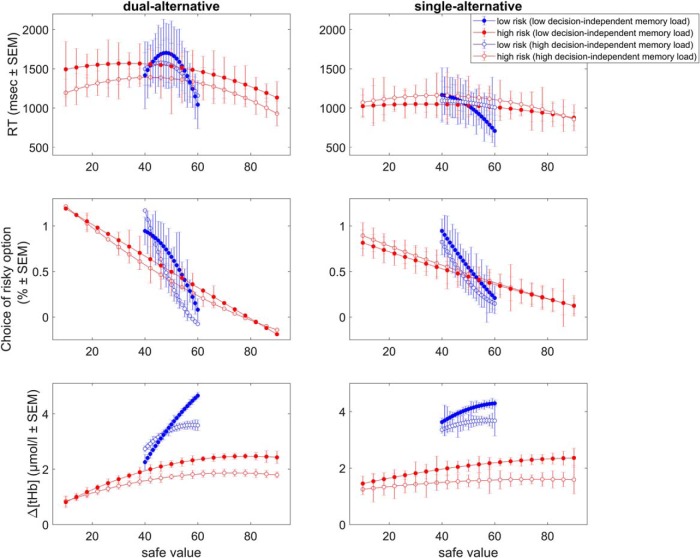
Slopes in Study 2. Top: RT slopes. Plots illustrate the addition of context-independent working memory load to the risk context and decision-related working memory dependence of RT, separately for the dual- and single-alternative experiments. Middle: Choice slopes. Plots illustrate the addition of context-independent working memory load to the risk context and decision-related working memory dependence of risky choice, separately for the dual- and single-alternative experiments. Bottom: Δ[tHb] slopes. Plots illustrate the addition of context-independent working memory load to the risk context and decision-related working memory dependence of Δ[tHb] response slopes averaged over all channels, separately for the dual- and single-alternative experiments. Note that all slopes are shown in terms of the coefficients for the second polynomial that best fitted the data (in a least-squares sense). Error bars represent SEM. See Tables 3 and 4 for statistics.

In Study 2, we assessed replicability of Study 1 and tested whether independent variation of working memory load in a secondary task had effects on neural value adaptation similar to having to remember one choice alternative. Study 2 (Fig. 8) revealed three main points. First, the behavioral data of Study 2 replicated those of Study 1 in that both RT (*load*, *F* = 2.291, *p* = 0.134; *context*, *F* = 9.407, *p* = 0.003; *experiment*, *F* = 0.844, *p* = 0.361) and choice behavior (*load*, *F* = 0.442, *p* = 0.508; *context*, *F* = 25.609, *p* < 0.001; *experiment*, *F* = 3.816, *p* = 0.058) revealed context-dependent effects, but no load- or experiment-dependent effects.

Second, the hemodynamic data also replicated those of Study 1 in that the PFC exhibited monotonic response increases with increasing safe values. These response increases occurred in both experiments of Study 2 and, just as in Study 1, were again steeper for the dual-alternative experiment than the single-alternative experiment (Fig. 8), indicating that context-related working memory demands reduce adaptive coding. In particular, slopes were again dependent on the risk context, with low-risk contexts eliciting steeper responses compared with high-risk contexts (Fig. 8), and the two divisive normalization parameters determining risk context-dependent differences were maximal activity (*A_max_*) and response slopes (σ; Tables 3 and 4).

**Table 3.**
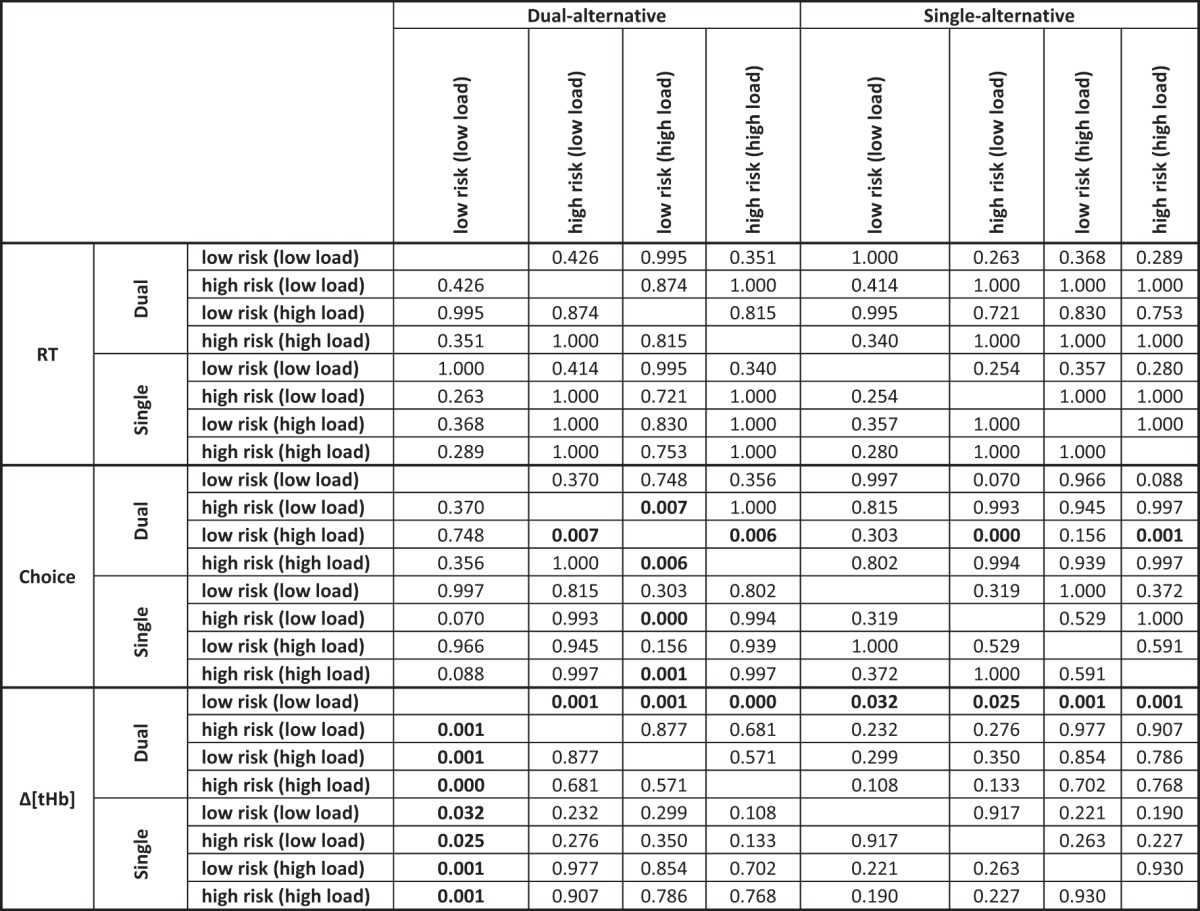
P-values of slope differences, Study 2

P-values for load- and risk-dependent differences (top left/bottom right quadrants of groups of 8 rows and 8 columns) and dual-alternative vs. single-alternative experiment differences (top right/bottom left quadrants) in regression coefficients relating RT, choice, and Δ[tHb] to safe values in each context, assessed using ANOVA with Bonferroni correction on the group level. Significant differences are bold (*p* < 0.05). See Fig. 8 for illustration.

**Table 4.**
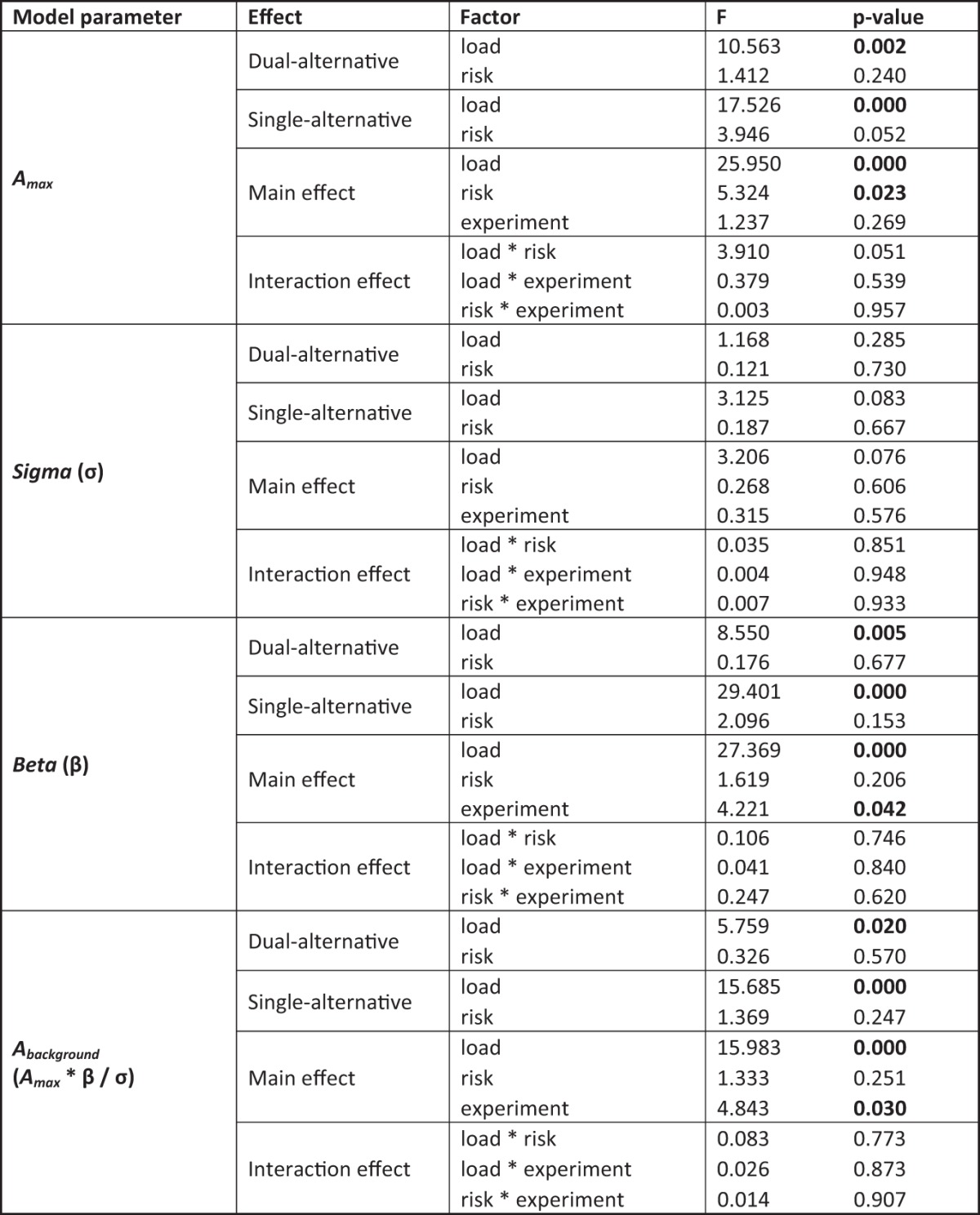
Comparison of model parameters, Study 2

ANOVA with within-subject factors Load (risk- vs. context-dependent demand on working memory) and *context* (low- versus high-risk), and between-subject factor *experiment* (dual- vs. single-alternative) to assess differences in the model 4 parameters (*A_max_* maximum activity, σ semisaturation, β baseline parameter). The ratio (*a_max_* * β/σ) can be summarized as predicted background activity (*A_background_*). Significant effects are bold (*p* < 0.05).

Third, after addition of the context-independent working memory load, neural activity in the dual-alternative experiment resembled neural activity in the single-alternative experiment. Thus, irrespective of whether working memory load was context-related (single-alternative experiment with high context-dependent working memory load) or not (dual-alternative experiment with no context-dependent working memory load), it reduced adaptive value coding (Fig. 8). As in Study 1, reduced normalization was associated with increased baseline pooled activity (β), which was the main critical parameter determining working memory–dependent differences in the steepness of response slopes, for both context-related and context-independent working memory load (Tables 3 and 4).

## Discussion

The present two studies examined the effect of working memory load on adaptive coding in the PFC using value normalization models in a risky decision-making context. We observed that the PFC encoded safe values in a context-dependent manner, such that hemodynamic response slopes were steeper for low-risk than high-risk contexts. Importantly, this pattern was significantly enhanced when risky alternatives were visually presented rather than maintained in working memory. Our results demonstrate that the PFC not only adaptively encodes value-related information in a context-dependent manner, but also that adaptation interacts with working memory demands that increase baseline activity and thereby reduce PFC capacity to normalize neural activity.

We formally compared four competing models of neural value adaptation (Louie et al., 2011). Please note that not all of these models used normalization to account for neural adaptation. Our findings suggest that the full divisive normalization model best accounts for neural value adaptation in PFC, even when penalizing for model complexity. With regard to that model, we show that working memory demand reduces capacity to suppress background activity, and we thereby provide a mechanistic account of how working memory interferes with value adaptation. Moreover, this finding suggests that participants actively maintained information in working memory throughout task blocks instead of selectively retrieving the information when it was needed.

Our data show that divisive normalization can be applied to risky decision-making in humans, further expanding the already wide range of applications of the model. In humans, divisive normalization has been applied not only to behavioral data on working memory (Bays, 2014), facial attractiveness processing (Furl, 2016), visual search (Miconi et al., 2015), visual masking (Tsai et al., 2012), and interocular suppression (Li et al., 2015), but also using fMRI with visual perception (Zhou et al., 2017), cross-orientation suppression (Brouwer and Heeger, 2011), and somatosensory processing (Brouwer et al., 2015). In the domain of value coding, divisive normalization has been applied less frequently, but general adaptation to context has been shown with fMRI in a variety of regions, including lateral (Akitsuki et al., 2003; Nieuwenhuis et al., 2005; Burke et al., 2016; Kirschner et al., 2016) and medial prefrontal cortex (Akitsuki et al., 2003; Nieuwenhuis et al., 2005; Elliott et al., 2008; Cox and Kable, 2014; Palminteri et al., 2015; Burke et al., 2016). In principle, divisive normalization could have been applied in many of these studies too. In any case, the wide range of applications reflects the notion that divisive normalization is a general organizing principle that captures adaptation in the entire neural machinery. The present study goes beyond that previous work in that we not only address divisive normalization with regard to the normalizing effects of risk contexts on the processing of safe values but also explore the impact of additional working memory demands. Methodologically, using fNIRS extends the examination of normalization using a different neuroimaging technique in humans.

### RT and choice behavior

Although RT and choice behavior in the two studies were sensitive to context, in contrast to PFC responses, they showed no effects of adaptation or working memory load. Previous research (Louie et al., 2013) revealed that divisive normalization can have behavioral effects. Differences in experimental paradigms may explain why the previous work was more sensitive for behavioral adaptation effects than our studies. Specifically, Louie et al. (2013) used choice situations with three alternatives and investigated the effect of varying the value of the least preferred alternative on choice between the two other, more preferred, alternatives. Their design therefore varied value within choice sets and examined choice behavior as a function of that manipulation, which changed the *V*s (i.e., the alternative values) that entered the denominator of the normalization equations (see “Model predictions” in Data Analysis). In contrast, we used choice situations with only two alternatives. Thus, in our design, value varied across rather than within choice sets, and changes in the *V*s were associated with different choice alternatives, which may explain why our design was less sensitive to behavioral adaptation effects. In any case, the fact that fNIRS picked up solid neural adaptation effects in the PFC in the absence of behavioral adaptation effects suggests that the neural findings cannot be explained by simple motor confounds reflecting behavioral adaptation effects.

### PFC encodes context-dependent value normalization

The PFC exhibited monotonic response increases as the value of safe alternatives increased (“Hemodynamic responses” in Results). These response increases were independent of actual choice and consistent with previous reports of PFC coding the value of safe alternatives (Tobler et al., 2009). The present data thus show that not only fMRI but also fNIRS can detect safe value coding in PFC hemodynamic responses.

Importantly, PFC responses showed significant adaptation of safe value signals driven by the risk context. In both experiments (and in both studies 1 and 2), we observed that the hemodynamic responses increased monotonically but differentially depending on the risk context (Fig. 3). In other words, a given increase in the magnitude of the safe alternative was represented by larger increases in PFC activity in low-risk than in high-risk contexts. Thus, it appears that the PFC encodes value not in an absolute manner (i.e., as it would have been predicted by the difference model or the basic divisive normalization model) but in a divisively normalized manner (i.e., as predicted by the enhanced and the full divisive normalization models; Louie et al., 2011; Carandini and Heeger, 2012). The two critical parameters determining these significant context-dependent differences were maximum pooled activity (*A_max_*) and response slope (σ; Fig. 7; Table 2). Thus, the adaptation to risky contexts can be captured with distinct parameters of normalization models originally described in the visual system.

### PFC encodes presence versus absence of risk context

Adaptive value normalization in PFC differentiated between the presence and absence of the risky alternatives. In line with our hypothesis derived from the animal literature on divisive normalization (Heeger, 1992; Carandini et al., 1997; Cavanaugh et al., 2002; Louie et al., 2011), this differentiation appeared to be related to reduced suppression of background activity (*A_Background_*). The critical parameter determining experiment-dependent differences in the steepness of response slopes (in both studies 1 and 2) was baseline pooled activity (β; Fig. 7; Table 2). Accordingly, presented risk (dual-alternative experiment) displayed robust context-dependent normalization as predicted by a full divisive normalization model with low baseline pooled activity. In contrast, remembered risk (single-alternative experiment) reduced the steepness of hemodynamic response slopes as predicted by the full divisive normalization model with high baseline pooled activity (Fig. 4). Entering these distinct values of baseline pooled activity in the fits of the full normalization model to the observed data fully captured the differences in slope steepness between the dual- versus the single-alternative presentation.

In other words, prefrontal hemodynamic activity appeared to integrate context-dependent information with the activity of neighboring prefrontal regions as captured by (*A_Background_*), a mechanism similar to that described in earlier work on in- and outside receptive field effects in sensory systems (Heeger, 1992; Carandini et al., 1997; Cavanaugh et al., 2002; Louie et al., 2011; Carandini and Heeger, 2012).

### Relation to attention and cognitive control

The PFC has been associated with a variety of functions other than working memory, such as attention and cognitive control (Duncan, 2001), which could potentially explain the presently observed normalization effects on value coding. Behaviorally, attention is typically associated with faster RT, and cognitive control is typically associated with slower RT (Nieuwenhuis and de Kleijn, 2013). Given that we found no significant RT differences between high and low working memory demand, the behavioral data provide little support for explanations in terms of attention or cognitive control.

Conversely, the two studies provide independent evidence that working memory load can affect neural value adaptation. In particular, a plausible interpretation for both studies might be that higher task-related or secondary working memory load had been processed in the prefrontal cortex, which amplified background activity, and subsequently resulted in a diminished suppression of the risk context-dependent adaptive steepening effects. A possible functional implication of lower background activity when working memory load is low may be that inhibitory mechanisms are working in the prefrontal network that preserve full normalization. In contrast, the reduced suppression of background activity when working memory load was high may suggest that the additional maintenance mechanisms diminished full normalization capacity through reduced baseline suppression. Speculatively, it is conceivable that the combination of high working memory capacity (Gathercole, 1999; Fry and Hale, 2000; Luna et al., 2004; Westerberg et al., 2004) and the ability to ignore interference (Tipper et al., 1989; Dempster, 1992; Ridderinkhof et al., 1997) facilitates divisive normalization.

Adaptation studies often use multiple stimuli or alternatives. The fact that the findings of Study 1 converged with those of Study 2 suggests that the more unusual presentation format of the single-alternative experiments is unlikely to explain the effects and that working memory load is the more relevant factor. Specifically, increasing working memory load through an independent secondary task in Study 2 reduced neural adaptation also in the more typical dual-alternative layout. In line with this finding, neural adaptation effects with single alternatives are not unprecedented (Tobler et al., 2005; Kirschner et al., 2016).

To apply previous work in the animal visual system (Louie et al., 2011), we treated the changing safe values in the current experiments as analogous to the changing visual stimuli inside the receptive field, whereas the risky alternatives were considered to be more constant, similar to extrareceptive field stimuli. However, this analogy should be handled very carefully because, in contrast to the visual experiments, the current experiments did not allow for a clear separation of in- and outside receptive field effects, as the safe and risky alternatives were inherently coupled in all trials (i.e., safe values were never shown without a presented or remembered risk context). Furthermore, whereas size and locations of receptive fields in visual cortex have been well defined (Spillmann, 2014; Spillmann and Dresp-Langley, 2015), receptive fields in prefrontal cortex are less well defined and involve complex combinations of visual, auditory, motor, memory, and emotional inputs (Rainer et al., 1998; Otani, 2007; Coward, 2013; Bullock et al., 2015).

Taken together, our results show that there is significant interaction between adaptive value coding and working memory. Although it is known that rewards can enhance access into working memory (Wallis et al., 2015), resulting in a reward-driven encoding bias, the opposite, i.e., a memory-driven value coding bias, has been considered primarily for episodic memory (Gershman and Daw, 2016). The current studies show that prefrontal suppression of background activity can be impaired by high working memory demands, resulting in diminished normalization. It remains to be seen whether reduced normalization capacity can explain noisier choice behavior, e.g., under dual-task conditions. Although we did not observe normalization-related changes in behavior with our relatively modest demand on working memory, one might expect that behavioral changes exist in patient populations. Indeed, lateral prefrontal value signals adapt less well in patients with schizophrenia compared with healthy controls (Kirschner et al., 2016). It is conceivable that tonic neurotransmitter levels (such as dopamine) may mediate between the working memory mechanisms necessary for assessing background reward contexts and concurrent accurate neuronal reward coding, a process that may be affected also in patients with addiction (Schultz, 2011).

### Methodological limitations

Task design may affect the exact nature of neural adaptation effects. For example, some previous studies focused on how neural signals encoding the value difference between two more preferred alternatives were affected by variations of a third, irrelevant, alternative. One such study (Chau et al., 2014) used fMRI and suggested that prefrontal context effects may not adhere to principles of divisive normalization. Specifically, it reported that value difference signals in the ventromedial PFC decreased in the presence of low-value third alternatives, with neural discrimination between the more preferred alternatives becoming harder. However, this finding contrasts with that of another study (Louie et al., 2013) that, in line with divisive normalization, showed that value difference signals increased in the presence of a low-value alternative. The designs of these two studies and of the present study differed in several respects, which could explain the differences. Specifically, the use of time pressure for decisions (Chau et al., 2014) could affect adaptation of decision-value signals differently than that of stimulus value signals, and the use of third alternatives (Chau et al., 2014; Louie et al., 2013) could facilitate the behavioral expression of adaptation as discussed above. Together, details in task design are important to consider when assessing neural adaptation effects.

It should be noted that the present model equations as adapted from Louie et al. (2011) were originally designed to predict the suppression of individual neurons based on a local network of neurons with receptive fields that either included or did not include a target. It may thus be asked whether the same equations can be applied to low-resolution fNIRS data that sum over metabolic activity within a large network of neurons, including both excitatory and inhibitory activity, presumably mediating the normalization. The translation is certainly not one-to-one, but it is conceivable that fNIRS is sensitive to the balance of excitation and inhibition within the cortex. This balance was originally thought to maintain the functional activity of tightly correlated cortical areas (Shadlen and Newsome, 1994; van Vreeswijk and Sompolinsky, 1996; Shu et al., 2003; Haider et al., 2006). Under certain forms of excitation/inhibition balance, average population activity has been recently suggested to scale with normalization-mediated changes in individual excitatory neuron firing rates (Rubin et al., 2015). This suggestion is in line with related computational work (Louie et al., 2014) using a simple differential equation model of a value normalization circuit, with both excitatory and inhibitory neurons. In particular, this work revealed that excitatory and inhibitory rates (at equilibrium) move together, implying that population-based measures such as fMRI or fNIRS could in principle be sensitive to inhibition-mediated normalized value coding.

Furthermore, it is conceivable that the present model specifications are rather imprecise. In the equations of divisive normalization we used (Louie et al., 2011), the value of the risky alternative is fixed to the expected value or average of the two outcomes, and the effects of manipulating risk context and working memory load were explained by the change of the fitted free parameters (maximal activity, response slope, and baseline activity). Specifically, because the risk context directly controls the composition of the values of the risky alternative, it is possible that the representation of the value of the risky alternative might be changed in different risk contexts (e.g., through additional noise). However, it is worth noting that the converging findings of Study 1 and Study 2 suggest that, at least for working memory load, these concerns seem to play a subordinate role. Still, future research may want to consider a wider range of model specifications.

Moreover, the potential cortical sources of the observed fNIRS signals are worth considering. We did not find significant differences in model fit between channels, which is in line not only with adaptation being a general principle that applies to both medial and lateral PFC but also with the low spatial resolution of fNIRS. However, it should be kept in mind that current evidence about prefrontal reward coding giving rise to within-context adaptation is limited even at the level of single neurons. Previously, we have shown with both fMRI (Tobler et al., 2009) and fNIRS (Holper et al., 2014) that lateral prefrontal activations increase with increasing safe rewards and with increasing subjective value of risky options. Relatedly, a larger number of single lateral prefrontal neurons respond to safe reward than to punishment (Kobayashi et al., 2006). However, the relative proportion of risk and safety coding neurons in different prefrontal regions is largely unknown, although single orbitofrontal cortex neurons appear to code value more commonly than objective risk (O’Neill and Schultz, 2010). Conversely, single anterior cingulate neurons represent reward probability to a greater extent than lateral prefrontal and orbitofrontal neurons (Kennerley et al., 2011). Yet, it remains unclear whether these relations hold also for other prefrontal regions and, if so, whether they result in different adaptation patterns. This issue is relevant because divisive value normalization is meant to apply to signals encoding the value of both safe and risky options.

In line with the single neuron findings mentioned above, our previous fMRI studies revealed objective risk signals in anterior cingulate cortex (Burke and Tobler, 2011) and subjective value signals in orbitofrontal cortex (Tobler et al., 2007). Still, relatively little is known about more dorsal prefrontal cortex, which we measured in the present fNIRS study. Neurons in supplementary motor regions show sustained prospective activity encoding the prediction of safe reward (Amador et al., 2000), whereas the well-known sustained retrospective activities of dorsolateral (e.g., Levy and Goldman-Rakic, 2000) as well as dorsomedial (e.g., Kaminski et al., 2017) prefrontal neurons represent the contents of working memory. In sum, it seems clear that the prefrontal regions we investigated encode the value at least of safe rewards, but more studies are needed to investigate the presence and relative prevalence of risk coding and the potential effects for neural adaptation. Importantly, our findings go beyond simple detection of neural value adaptation by characterizing one plausible implementation mechanism through formal comparison of competing models of value adaptation.

## Conclusion

The present studies showed that in a risky decision-making context adaptive value coding in the PFC can be captured with divisive normalization models. In both studies, adaptation was more pronounced during low-risk compared with high-risk contexts, and when working memory load was low compared with when it was high. These effects were reflected in increased background activity (reduced background suppression) when working memory load was high. Our data reinforce the ubiquity of adaptive coding, elucidate its dynamic nature, and reveal that subtle changes in working memory load can influence the degree of neural value adaptation.

## Synthesis

Reviewing Editor: Morgan Barense, University of Toronto Decisions are customarily a result of the Reviewing Editor and the peer reviewers coming together and discussing their recommendations until a consensus is reached. When revisions are invited, a fact-based synthesis statement explaining their decision and outlining what is needed to prepare a revision will be listed below.

The authors effectively addressed some of the concerns, but the primary concern (Comment 1) requires a more direct response to justify the methodological approach. Specifically, the equations that the authors adapted from Louie et al (2011) were designed to predict the suppression of individual LIP neurons based on the local network of neurons with receptive fields that either included or did not include a target. We are concerned that the authors are making a big leap when they apply these same formulas to fNIRS data. This low-resolution data is summing over metabolic activity within a large network of neurons, including both excitatory responses and the inhibitory activity that is presumably mediating the normalization. Should the same formulas be used to predict fNIRS as single unit data in this case?

The authors should directly state their logic to justify why these models should predict hemodynamic activity across large patches of cortex. Caveats on their interpretations should be directly acknowledged where appropriate.

Additionally, the authors imply that assessing divisive normalization using fNIRS/fMRI is relatively well established and that their primary advance is to assess how memory influences, not to validate the approach. But how well established is the approach in the first place? Many of the included citations are for behavioral studies in humans, electrophysiology studies in animal models, or fMRI/fNIRS studies looking at value coding in humans. We feel that the authors should specifically discuss prior research that has used either fMRI or fNIRS to study divisive normalization, and they should be clearer regarding how their approach compares to this published work.

Minor comment: the revised manuscript claims “Moreover, the fact that in the present study fNIRS detected neural adaptation in the absence of behavioral adaptation effects supports the sensitivity of fNIRS as a measure of neural activity.” This is a very generous spin on a null behavioural finding. It is also possible that fNIRS is not effectively measuring divisive normalization. Please remove this line.
